# Islands and hybrid zones: combining the knowledge from “Natural Laboratories” to explain phylogeographic patterns of the European brown hare

**DOI:** 10.1186/s12862-019-1354-y

**Published:** 2019-01-10

**Authors:** Themistoklis Giannoulis, Dimitrios Plageras, Costas Stamatis, Eleni Chatzivagia, Andreas Tsipourlianos, Periklis Birtsas, Charalambos Billinis, Franz Suchentrunk, Zissis Mamuris

**Affiliations:** 1 Laboratory of Genetics, Comparative and Evolutionary Biology, University of Thessaly, Viopolis, Mezourlo, Larissa, Greece; 2 Department of Forestry and Natural Environment Administration, TEI of Thessaly, Larissa, Greece; 3 Faculty of Veterinary Medicine, University of Thessaly, Karditsa, Greece; 4 Research Institute of Wildlife Ecology, University of Veterinary Medicine Vienna, Vienna, Austria

**Keywords:** Adaptive variation, Neutral loci, Brown hare, Phylogeography

## Abstract

**Background:**

The aim of the study was to use hybrid populations as well as island populations of the European brown hare (*Lepus europaeus)* to explore the effect of evolutionary events, such as the post-deglaciation translocations, spontaneous and human-mediated, local adaptation and the genetic drift in the shaping of the phylogeographic patterns of the species. For this purpose, we used molecular markers, both nuclear and mitochondrial, that are indicative for local adaptation as well as neutral markers to elucidate the patterns of population differentiation based on geographic isolation and the clade of origin. To broaden our analysis, we included data from our previous studies concerning mainland populations, to explore the genetic differentiation in the base of the geographic origin (mainland/island) of the populations.

**Results:**

Our results suggest that local adaptation shapes the differentiation in both genomes, favoring specific alleles in nuclear genes (e.g. DQA) or haplotypes in mtDNA (e.g. Control Region, CR). mtDNA variation was found to be in a higher level and was able to give a phylogeographic signal for the populations. Furthermore, the degree of variation was influenced not only by the geographic origin, but also by the clade of origin, since specific island populations of Anatolian origin showed a greater degree of variation compared to specific mainland populations of the European clade. Concerning the hybrid population, we confirmed the existence of both clades in the territory and we provided a possible explanation for the lack of introgression between the clades.

**Conclusion:**

Our results indicate that the Quaternary’s climatic oscillations played a major role in the shaping of the phylogeographic patterns of the species, by isolating populations in the distinct refugia, where they adapted and differentiate in allopatry, leading to genome incompatibilities observed nowadays.

**Electronic supplementary material:**

The online version of this article (10.1186/s12862-019-1354-y) contains supplementary material, which is available to authorized users.

## Background

By their own very nature, islands and hybrid zones are considered as “natural laboratories” for the study of the acting evolutionary forces in populations inhabiting such territories [1, 2]. Island populations are often small, derived from a bigger continental population through the founder effect, resulting in reduced levels of heterozygosity [3]. Migrations to and from the population are usually rare, especially for species with low dispersal ability. Thus, genetic drift is acting to shape the levels of variation through generations. Translocations of such populations are usually human-mediated, especially when the species are flightlesss (e.g. the paradigm of Iberian red deer, [4]) but many times, permanent or transient land bridges mediate the translocations [5, 6]. On the other hand, hybrid populations are formed in territories when two distinct genomes meet and hybridize [7]. During the last ice ages of the Quaternary, populations of the North have declined dramatically by extinction or by migration to southern and warmer territories [8, 9] (e.g. the example of the Gray Wolf, [10]). In the case of Europe, northern populations have migrated to the South, where certain and distinct refugia for the species have been recognized so far in Italy, the Iberian Peninsula, the Balkans and Anatolia [8, 11–13]. The isolation of those populations for a long period resulted in their genetic differentiation. Subsequent post-glacial expansion of the refugial populations led to the formation of the hybrid zones, where different genomes met, yet their differences were responsible for hybrid unfitness [9]. Genetic differentiation of the genomes could be a result of selection or drift. Despite hybridization, the intraspecific lineages do not geographically mix, which could be a result of the classic hybrid breakdown: F1 hybrids are usually fertile and well-adapted but F2 hybrids tend to show low levels of fitness and adaptation [2, 14, 15]. It is believed that hybrid zones are maintained by the action of two different processes: random dispersal and selection against hybrids [7]. The reduced fitness of the hybrids is usually caused by many genes with small effect, spreading throughout the genomes rather that a big contribution of a few genes [2]. So, alleles that are crossing the zone are negatively selected: they are in the wrong environment or they are combined with wrong alleles [7]. These parapatric populations and the hybrid zones may reflect the different stages of speciation [2]. The existence of hybrid zones has been well characterized for a variety of species, such as the European grasshopper (*Chorthippus parallelus)*, the European green toad (*Bufo viridis)*, the bicolored shrew (*Crocidura leucodon)*, the crested newt (*Triturus karelinii)* and the European brown hare (*Lepus europaeus)* (reviewed in [16]).

A model species to study the evolutionary forces acting on multiple levels of population dynamics is the European brown hare (*Lepus europaeus,* Pallas). Brown hare distribution covers Europe and Anatolia (which is a part of modern Turkey) and has been introduced in the past to South America, Australia and New Zealand [17]. It is also present in European islands, such as the UK and Mediterranean islands. The introductions of the species to these islands have followed two major paths: (a) human mediated translocations, for restocking operations for hunting purposes or as a symbol of fertility, brought during migration activities and/or (b) by movement through temporary land bridges that connected the mainland with these islands and gave the ability to migrate [18]. Thus, hares from islands with proximity to the Anatolian coast, like Chios or Lesvos originated from the Anatolia and migrated when the sea level was lowered enough during the Late Glacial Maximum [19, 20].

**Fig. 1:**
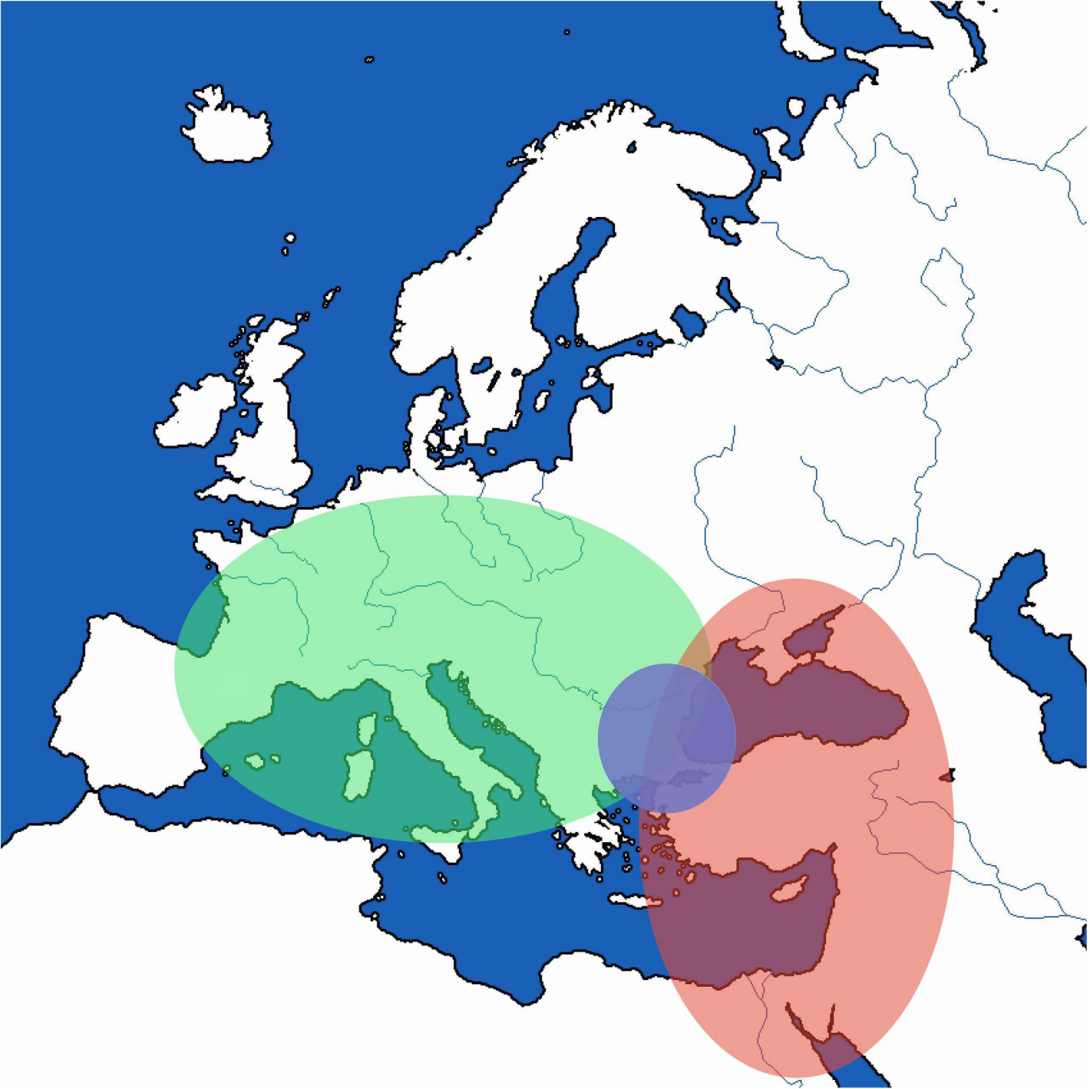
The distribution of the species of *Lepus europaeus*. The discrete clades (green for the European, red for the Anatolian) are having a contact zone in North-Eastern Greece and Bulgaria (blue)

Two major studies for the phylogeography of the species of *L. europaeus* have revealed the existence of two well-separated, spatially and genetically, clades: The European clade, which can be subdivided in Central European and South-Eastern European sub-clade, and the Anatolian clade [20, 21]. The European clade is present in European countries, such as Germany, France, Italy, Greece etc., and the Anatolian clade encompasses Anatolia, Cyprus, Israel and the Mediterranean islands with proximity to the Anatolian coast. Despite the distinct geographical distribution of each clade, there is a contact zone in Northern Greece and Bulgaria where the two clades meet and form a hybrid zone [20, 21]. The distribution of the clades is shown in Fig. 1. A recent study of the population of the hybrid zone has revealed that the intruding clade is the Anatolian and also areas of genetic discontinuities were found, indicating the reduced gene flow between clades [22]. The reduced gene flow between the clades on contact zone may have resulted due to the absence of geographical mix of the clades anywhere else than the hybrid zone; no individuals have been found carrying the European haplotype in Anatolia and vice versa [21, 22]. Djan and her colleagues [23] by using an extensive sampling in the Balkans (Serbia, Kossovo, Bulgaria, FYROM) analysed a partial Control Region sequence of the mitochondrial DNA and proposed a refined phylogeographic model for the species, which is in accordance with the previous ones, while they also observed the lack of introgression of the Anatolian clade further from the contact zone in Bulgaria and Northern Greece.

In two recent studies, we tried to explain this lack of reciprocal introgress ion by using two different approaches. In the first one [24], we used transcriptomics data from individuals of both clades and detected signs of co-adaptation among the nuclear genes of OXPHOS and their respective mitochondrial counterparts, which cooperate to perform the cellular respiration. According to the theory of co-adaptation, genes that cooperate to complete a process are thought to have similar evolutionary rates [25]. In the second study [26] we sequenced 14 complete mitochondrial genomes from both clades. The differentiation of the genes encoding for the mitochondrial OXPHOS subunits was confirmed once again. Thus, the contribution of “mother’s curse” could be responsible for the hybrid breakdown in the hybrid zone: male individuals carrying a specific mitochondrial haplotype showed a lower reproductive ability. The matrilineal inheritance of cytoplasmic genetic elements, such as the mtDNA, can lead to a sex-biased selection, that favors beneficial mutations for the female individuals, while they may have a negative impact in male’s fitness [27, 28]. Sperm cells have higher energy demands compared to ova, so when the mitochondrial DNA has to cooperate with a differentiated genetic background, there is a breakdown in energy production, affecting the male individuals rather than the females [27]. Evidence for this phenomenon has been found in a variety of species, including insects [29], chickens [30] as well as humans [31, 32]. Moreover, the phenomenon has been documented in the species of the brown hare, in individuals kept in captivity, where crosses between individuals with different mitochondrial background resulted in different reproductive success [15].

This model of differentiation is in accordance with the allopatric model, which requires a geographic gap between the populations and a reduced gene flow between them for a long period of time. These criteria are met by the conditions that existed during the Ice Ages. Separated populations have developed local adaptations and have diversified through time and these have resulted in a genetic incompatibility, when these two genomes meet and hybridize.

In the context of adaptive variation, one of the loci widely studied is the major histocompatibility complex (MHC). MHC is part of the adaptive immune system and its gene family is comprised by several genes, usually organized in clusters (e.g. in human chromosome 6, in mouse chromosome 17, data from Ensembl Genomes (http://ensemblgenomes.org/info/genomes)). The major role of MHC is the presentation of processed antigens to T-cells (CD4 or CD8). MHC molecules are binding antigens using specific structural domains, called peptide-binding regions (PBRs) or binding pockets. A match between the three components, PBR, the antigen and receptors of T-cell are required to start the immune response of the organism [33]. MHC gene family possesses a position among the most polymorphic genes and most of the variation is mapped in the PBR regions, resulting in the extended ability of recognizing a great repertoire of antigens and subsequent presentation to T-cells [34]. In such a manner, populations are capable of resisting a variety of pathogens, affecting populations’ adaptation and viability. These high levels of variation are maintained by overdominance or frequency-dependence [35, 36]. Generally, the variation in MHC genes is maintained by positive selection, increasing the available pool of different antigen presenting molecules [37, 38].

Many associations between the variation of DQ genes (genes of the MHC class II complex, encoding for surface receptor proteins found on antigen presenting cells) and susceptibility or resistance to disease have been conducted so far, reflecting the importance of maintaining high levels of variation at these particular genes. In the species of *Lepus europaeus*, there are paradigms correlating specific alleles of MHC genes with susceptibility to European Brown Hare Syndrome (EBHS) virus [39]. Furthermore, studies in other vertebrates have associated MHC variations with susceptibility to autoimmune diseases, individual odors, mating preferences, kin recognition, co-operation and reproduction success [40–42]. Especially in the species of *L. europaeus*, the balance of the energy allocation to pathogen resistance and development is critical, due to their fast growth rate [43]. The European brown hare is the only small or medium sized mammal in Europe which raises their young above the ground right from the day of birth, with a little protection thereafter [44, 45]. Considering the energy costs for the fast development, there is a need for a very efficient immune system, to ensure the allocation of sufficient energy to their fast growth rather than to pathogen resistance [46].

The aim of this study is to address the contribution of adaptive selection and the genetic drift in the shaping of phylogeographic patterns and population structures observed in the species. For this purpose, we focused on the adaptive and neutral variations that are established in island populations of the species of *L. europaeus* and compare it with the respective levels in mainland populations. For adaptive variation, we use a marker of the MHC gene family and more specifically the exon 2 of DQA gene (MHC class II), that has been proven to be useful for this kind of studies [33, 39] and a mitochondrial region [containing partial sequence of the CytB gene, two genes encoding for tRNAs (tRNAThr and tRNAPro) and a partial sequence of Control Region (CR) or D-Loop]. For the study of non-adaptive variation, we genotyped six microsatellite loci. Additionally, we applied the same microsatellite markers in a hybrid population and continental populations that belong to the major haplogroups, to estimate the current population dynamics in this particular area and how it is affected by the simultaneous existence of the two diversified mitochondrial haplotypes in the same region.

## Results

### Sample assignment

All samples were assigned to the 3 major phylogeographic groups according to their restriction pattern of the partial CytB gene according to the method proposed by Stamatis and his colleagues [47]. The samples from Greece were assigned to the South-Eastern European group, while the samples of New Zealand and the UK were assigned to the Central European group. The samples of the Greek islands along with the samples of Anatolia, Israel and Cyprus were assigned to the Anatolian group, with only one exception in the Cypriot population (1 sample was assigned to the Central European group). The samples from the hybrid zone were assigned as follows: 6 were assigned to the SE European, 20 to the Central European and 22 to the Anatolian group.

### Variation in MHC DQA exon 2

Twelve different alleles (named allele 1 – allele 12) were detected combined to 21 different genotypes. Six alleles were discovered for the first time and were deposited in GenBank under the accession numbers MH029632-MH029637. Allele frequencies are presented in Table 1. As shown in the table, there are overlapping alleles between populations belonging to different clades (e.g. alleles 1 and 2). Pairwise *F*_st_ values of the population pairs range from 0.046 (Rodos vs New Zealand) to 0.434 (Samos vs New Zealand) (Table 2).

**Table 1:** Allele frequencies (%) of DQA exon 2

	1	2	3	4	5	6	7	8	9	10	11	12	Samples
N.Zealand	88.2	3.9	5.3	0.0	2.6	0.0	0.0	0.0	0.0	0.0	0.0	0.0	38
Samos	1.4	50.0	0.0	4.3	0.0	0.0	0.0	0.0	0.0	44.3	0.0	0.0	35
Rodos	66.7	0.0	0.0	0.0	0.0	0.0	0.0	16.7	16.7	0.0	0.0	0.0	6
Mytilini	0.0	41.7	25.0	0.0	0.0	0.0	0.0	33.3	0.0	0.0	0.0	0.0	12
Chios	11.8	8.8	0.0	5.9	0.0	0.0	0.0	5.9	61.8	0.0	5.9	0.0	17
Cyprus	0.0	17.5	2.5	0.0	0.0	25.0	48.8	0.0	0.0	0.0	0.0	6.3	40

**Table 2:** Pairwise F_st_ values of DQA exon 2 genotype frequencies

	N.Zealand	Samos	Rodos	Mytilini	Chios
Samos	0.43				
Rodos	0.05	0.18			
Mytilini	0.38	0.11	0.21		
Chios	0.39	0.23	0.15	0.20	
Cyprus	0.38	0.20	0.13	0.12	0.19

### Pocket analysis

The translation of the sequences of the 12 alleles also revealed 12 different amino acid sequences, which means that each allele carries at least one different non-synonymous mutation compared to the rest. Specific positions of the amino acid sequence encode for partial regions of pocket 1, 6 and 9 according to Bondinas and his colleagues [48]. In this study, we detected five, four and three different pocket variants for pocket 1, 6 and 9 respectively. Their frequencies are presented in Additional file 1: Table S3. Several variants were present only in one or two of the studied populations. Particular variants were detected in Cyprus and Chios populations, while being absent in their parental populations of Anatolia. All pocket variants detected in this study were also discovered in the study of Koutsogiannouli et al. [28]. The unique pocket combinations of the alleles (P1-P6-P9) were 8 and their frequencies are presented in Table 3.

**Table 3:** Unique pocket combinations and their respective frequencies (%)

Pockets Combinations	N.Zealand	Samos	Rodos	Mytilini	Chios	Cyprus
YHEFWR/NETAN/YNILR	92.11	51.43	66.67	41.67	20.59	17.50
YHLFWT/NETAN/YNILR	5.26	0.00	0.00	25.00	0.00	2.50
YHQFWT/NNTAN/YNILR	0.00	4.29	0.00	0.00	73.53	0.00
YHEFWA/NNTAN/YNIMR	2.63	0.00	16.67	0.00	0.00	0.00
YHQFWA/NNTEN/YNILR	0.00	0.00	0.00	0.00	0.00	25.00
YHQFWA/NNTAG/YGIMR	0.00	0.00	0.00	0.00	0.00	55.00
YHQFWT/NNTAN/YNIMR	0.00	0.00	16.67	33.33	5.88	0.00
YHQFWA/NNTAN/YNILR	0.00	44.29	0.00	0.00	0.00	0.00

The most frequent haplotype in both clades was found to be the YHEFWR/NETAN/YNILR with a significant difference in their frequency (92% vs 34%). Moreover, three unique combinations were revealed in the Anatolian group, two of them only present in Cypriot population (YHQFWA/NNTAG/YGIMR and YHQFWA/NNTEN/YNILR) and the third one (YHQFWA/NNTAN/YNILR) solely in Samos.

### Selection analysis

A total of 49 MHC DQA exon 2 sequences (6 new alleles and 43 already deposited in GenBank) were used for the selection analyses. Since we used more than one methods for detecting selective pressures, we chose sites that were in accordance with at least two of them. Applying the above criteria, we found strong evidence for positive selection in codon 8 and six positions under negative selective pressure (codon positions: 2, 10, 17, 19, 26, 30). Neither the positively selected site nor the negatively selected sites encode for the PBR regions that we mentioned above.

### Mitochondrial DNA analysis

After splitting the region in its counterparts, we detected 10 different haplotypes for the tRNAs genes, 23 for the partial CytB gene and 54 in the partial CR sequences (GenBank Accession Numbers: MH029638-MH029691). The outcome of the BlastClust program showed that the CytB and tRNAs regions fail to show geographic grouping of the sequences in finer scale (sequences from different populations were grouped together due to their 100% similarity) while CR grouping had a clear geographic signal following the distribution of the samples. The only exception in the CR grouping were three shared haplotypes between England and New Zealand (possibly due to their common origin), which led us to treat these two populations as one in the analysis. The pairwise genetic distances among the CR haplotypes are shown in Table 4. Pairwise distances varied from 0.65% (between Samos and Lesvos) to 6.4% between England/New Zealand and Chios. The overall mean distance was calculated to be 3.7%, while the intragroup distances ranged from 0.22% (Chios) to 2.55% (Anatolia). Island populations had an average value of intrapopulation genetic distance equal to 0.63% and the continental population had 2.55% respectively. By assigning the populations to the two major clades, we calculated an intragroup distance of 0.58% for the European clade and 2.7% for the Anatolian, while the pairwise distance between them was calculated at 5.97%.

**Table 4:** Pairwise distance of CR haplotypes per population

	Rhodes	Anatolia	Lesvos	Samos	Chios	Cyprus
Anatolia	2.9					
Lesvos	2.3	2.2				
Samos	2.4	2.1	0.7			
Chios	2.7	2.9	2.9	2.9		
Cyprus	4.0	3.2	3.1	3.1	4.0	
UK/N. Zealand	6.0	5.9	5.5	5.9	6.4	5.9

**Fig. 2:**
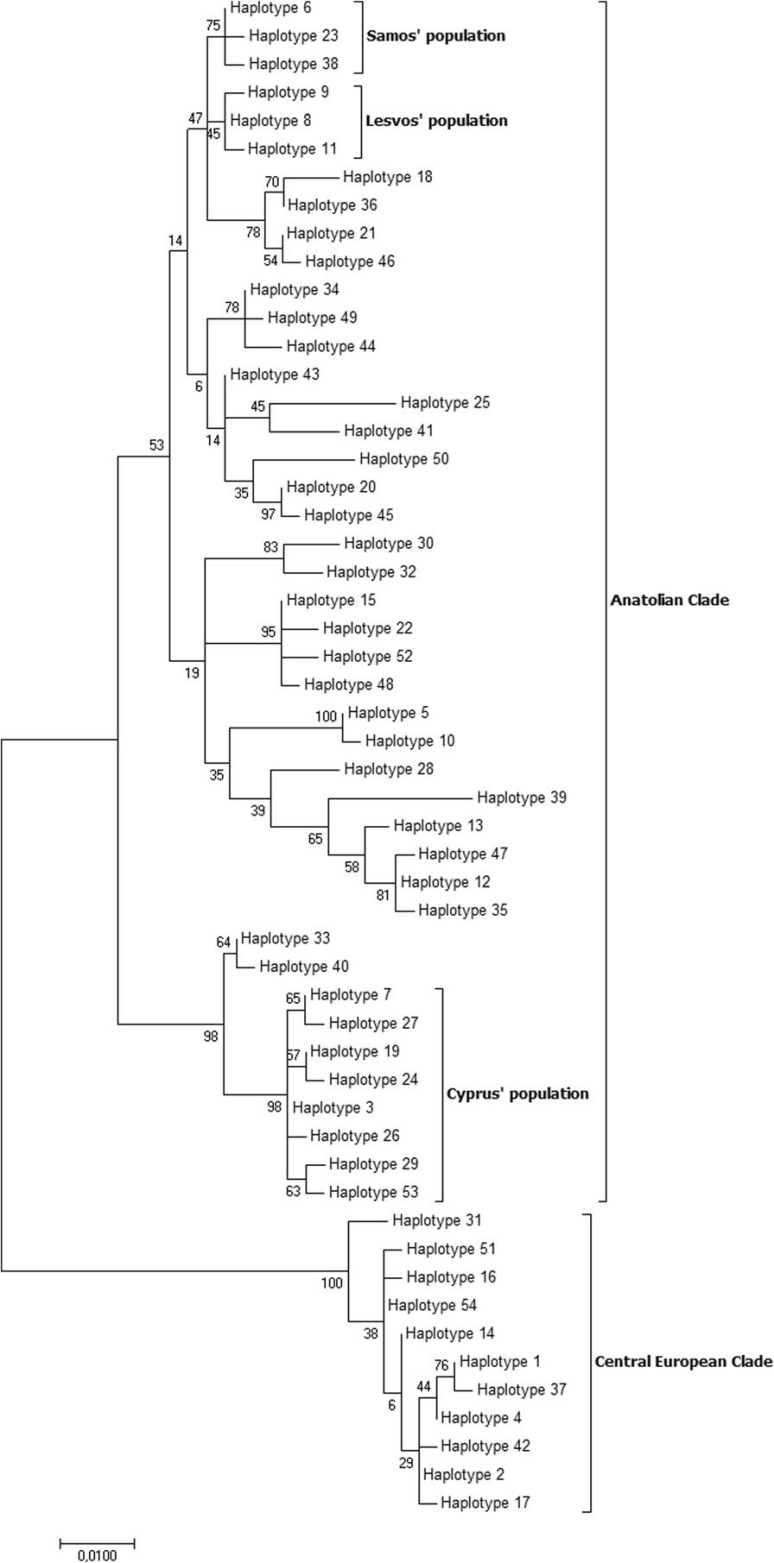
An ML tree of the unique CR haplotypes

**Fig. 3:**
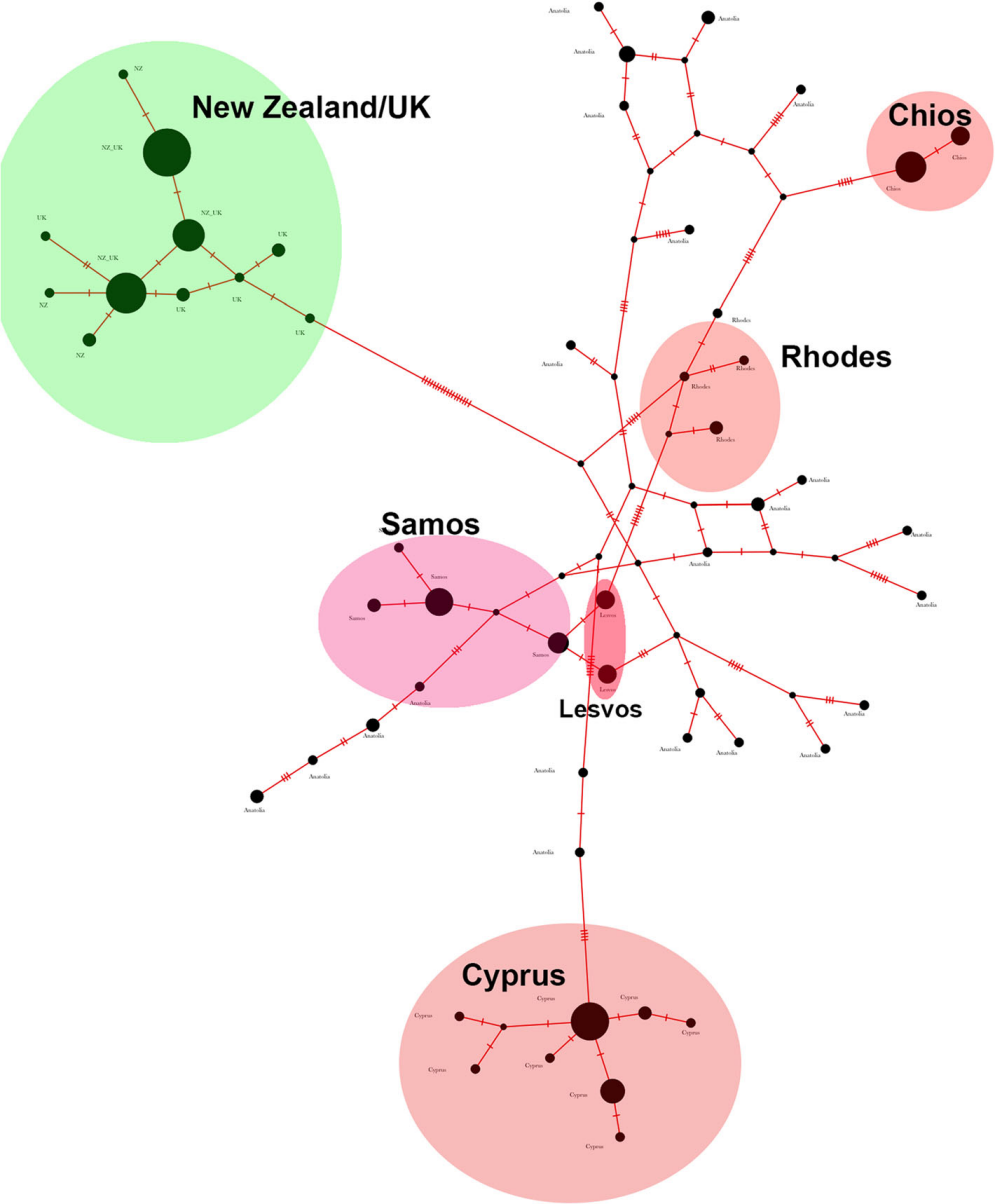
Network analysis of the CR haplotypes. Coloring is in accordance with the clade of origin of the population (green for the European, red for the Anatolian)

The phylogenetic tree (Fig. 2) revealed the expected topology; we observed two major groups corresponding to the two major clades, the European and the Anatolian. In the Anatolian clade, there are also two sub-clades formed, one containing the haplotypes of Cyprus mainly and the other containing the rest of Anatolian haplotypes. Moreover, the haplotypes of Samos and Lesvos are grouped together in the sub-structuring of the Anatolian clade. This type of grouping for the samples of Cyprus, Samos and Lesvos is in accordance with the results from the network analysis in the study of Kasapidis and his colleagues [20] and with the results of our network analysis (Fig. 3).

### Microsatellite analysis

#### Tests for HWE and LD

By comparing the results of the chi-square tests with the corrected *p*-values using the sequential Bonferroni method, we detected population-locus specific deviations from the HWE (Additional file 2: Table S1). In most of these cases, the corresponding Fis values for these combinations were positive, thus were indicative for a deficiency in heterozygotes. Furthermore, in these specific combinations of populations and loci, there was an excess of null alleles (e.g. 13% in Cyprus-Sol30, 21.7% Cyprus-Sat2) and a significantly large frequency of rare alleles (frequency < 10%) (e.g. 19.5% of Cyprus-Sat2, 28.5% of UK-Sat2 were assigned in rare alleles combined).

All 15 tests for LD failed to reject the null hypothesis of linkage equilibrium between the pairs of loci (Additional file 3: Table S2). By using the nominal p-value for our comparisons, the pair of loci Lsa1 and Sat2 showed a significant signal of LD. However, the markers are mapped in chromosomes 5 and 15 of the rabbit genome respectively (which is the most relative species to *L. europaeus* sequenced so far), so we believe this is not a true signal of physical linkage of the loci. Based on these results, we used all the 6 markers for the downstream analysis.

#### First approach– Population number = 11

The six microsatellite loci analyzed in this study were polymorphic with a mean number of alleles per locus equal to 15.3, ranging from 6 (Lsa6) to 30 (sol30). The number of alleles, the observed and the expected heterozygosities (H_obs_ and H_exp_ respectively) calculated for each locus and population are shown in Additional file 1: Table S3. The continental populations had an average of H_obs_ equal to 0.52 while the island populations had an average of 0.30. Continental populations are shown to contain approximately double numbers of alleles compared to the island populations (6.6 vs 3.2 in Sol08, 11 vs 4.1 in Sol30, 4.5 vs 2.85 in Sol 33, 5 vs 4.7 in Lsa1, 2.75 vs 1.85 in Lsa6 and 10.25 vs 5.28 in Sat2). The number of alleles and the H_obs_ and H_exp_ for each locus and population are presented in Additional file 4: Table S4.

Pairwise *F*_st_ values (Table 5) ranged from 0.05 between N. Zealand and the UK, (Central European clade), to 0.44 between Samos and Mytilini (Anatolian clade). The hybrid zone population showed a minimum pairwise value compared to the Greek population (0.07) and the maximum values were calculated when compared with Anatolian populations (0.27 vs Mytilini, 0.26 vs Rodos, 0.24 vs Anatolia).

**Table 5:** Pairwise F_st_ values (k = 11)

	Greece	N. Zealand	UK	Rodos	Mytilini	Samos	Anatolia	Chios	Cyprus	Israel	Hybrid Zone
Greece		0.22	0.15	0.17	0.22	0.20	0.17	0.18	0.17	0.16	0.07
N. Zealand	0.22		0.05	0.28	0.37	0.33	0.37	0.36	0.29	0.38	0.22
UK	0.15	0.05		0.23	0.27	0.17	0.28	0.29	0.23	0.30	0.13
Rodos	0.17	0.28	0.23		0.31	0.40	0.22	0.23	0.15	0.21	0.26
Mytilini	0.22	0.37	0.27	0.31		0.44	0.33	0.36	0.26	0.35	0.27
Samos	0.20	0.33	0.17	0.40	0.44		0.31	0.38	0.31	0.38	0.14
Anatolia	0.17	0.37	0.28	0.22	0.33	0.31		0.24	0.16	0.21	0.24
Chios	0.18	0.36	0.29	0.23	0.36	0.38	0.24		0.21	0.30	0.23
Cyprus	0.17	0.29	0.23	0.15	0.26	0.31	0.16	0.21		0.07	0.20
Israel	0.16	0.38	0.30	0.21	0.35	0.38	0.21	0.30	0.07		0.21
Hybrid Zone	0.07	0.22	0.13	0.26	0.27	0.14	0.24	0.23	0.20	0.21	

**Fig. 4:**
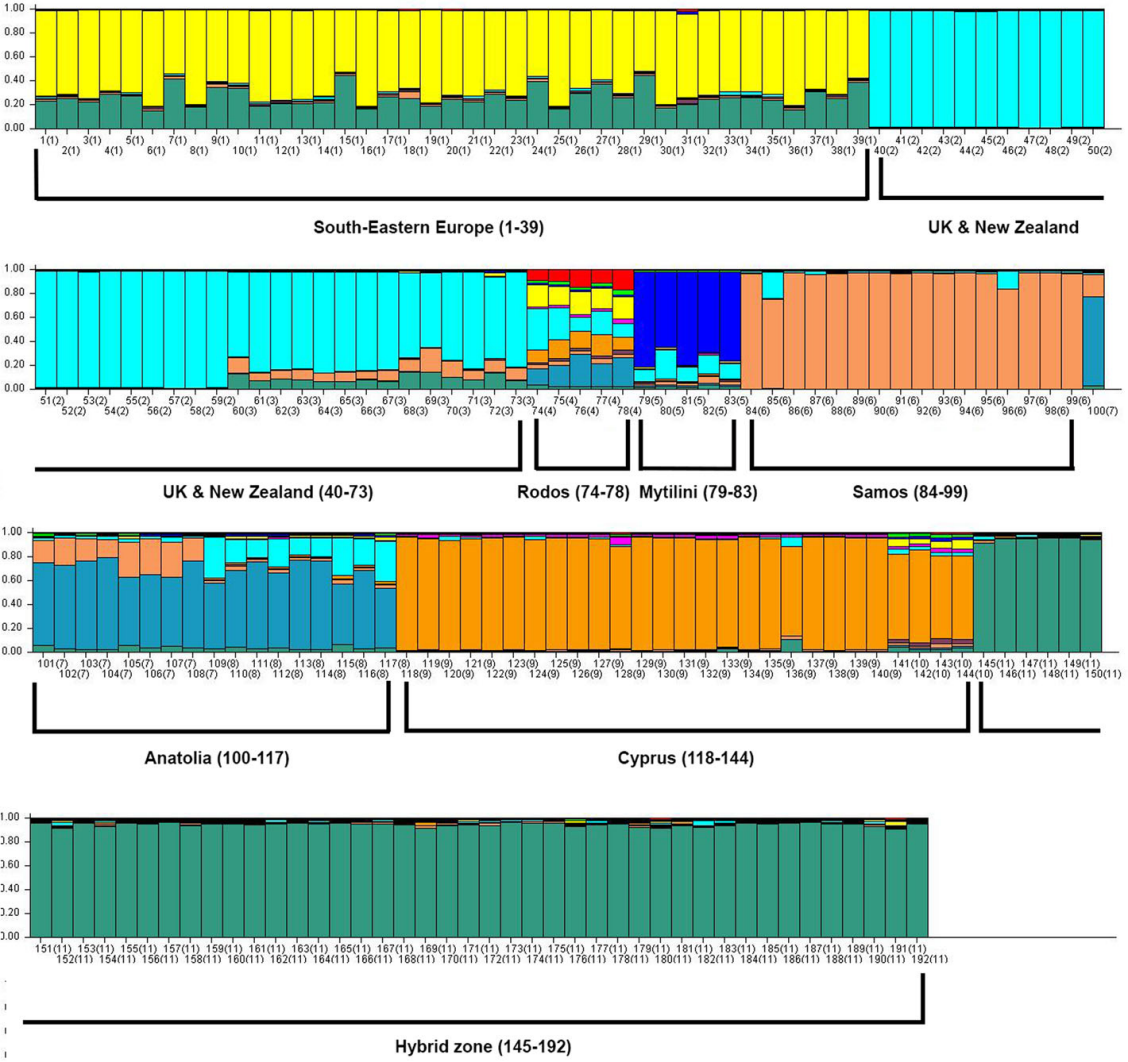
Samples’ assignments to eleven clusters, using the geographic origin of the samples

Plotting the q-values of each individual (Fig. 4) revealed differences between the populations of the three haplogroups (South-Eastern European group: samples 1–39, Central European Group: samples 40–73, Anatolian Group: Samples 74–144), with the populations of Rodos (74–78) and Mytilini (79–83) showing an admixture of patterns. The populations of Samos, Anatolia and Cyprus (84–99, 100–117 and 118–144 respectively), despite belonging to the same clade, are shown to have diversified from each other. The hybrid population (145–192), shows a uniform pattern, regardless of the mitochondrial lineage of origin. The same pattern is observed partially in the Greek individuals also.

#### Second approach – Population number = 3

Examining the plot K~ΔΚ, we could infer the real value of K for our data to be equal to 3 as expected, due to the phylogeographic status of the species. We sub-divided the samples to the 3 groups using the q-values, which are calculated automatically by the Structure program. Samples from Greece and the hybrid zone were assigned to group 1, samples from N. Zealand and England were assigned to group 2 and the Anatolian samples were grouped in group 3 with minor exceptions (e.g. the Cypriot sample carrying the Central European mitochondrial haplotype). Pairwise *F*_st_ values were recalculated (Table 6) for all the three population pairs.

**Table 6:** Pairwise F_st_ Values (k = 3)

	Anatolian	Central European	SE European
Anatolian		0.19	0.13
Central European	0.19		0.11
SE European	0.13	0.11	

The observed and expected heterozygosities (H_obs_ and H_exp_ respectively) were calculated for each locus and population. The H_obs_ values ranged from 0.04 to 0.88 (Additional file 5: Table S5).

**Fig. 5:**
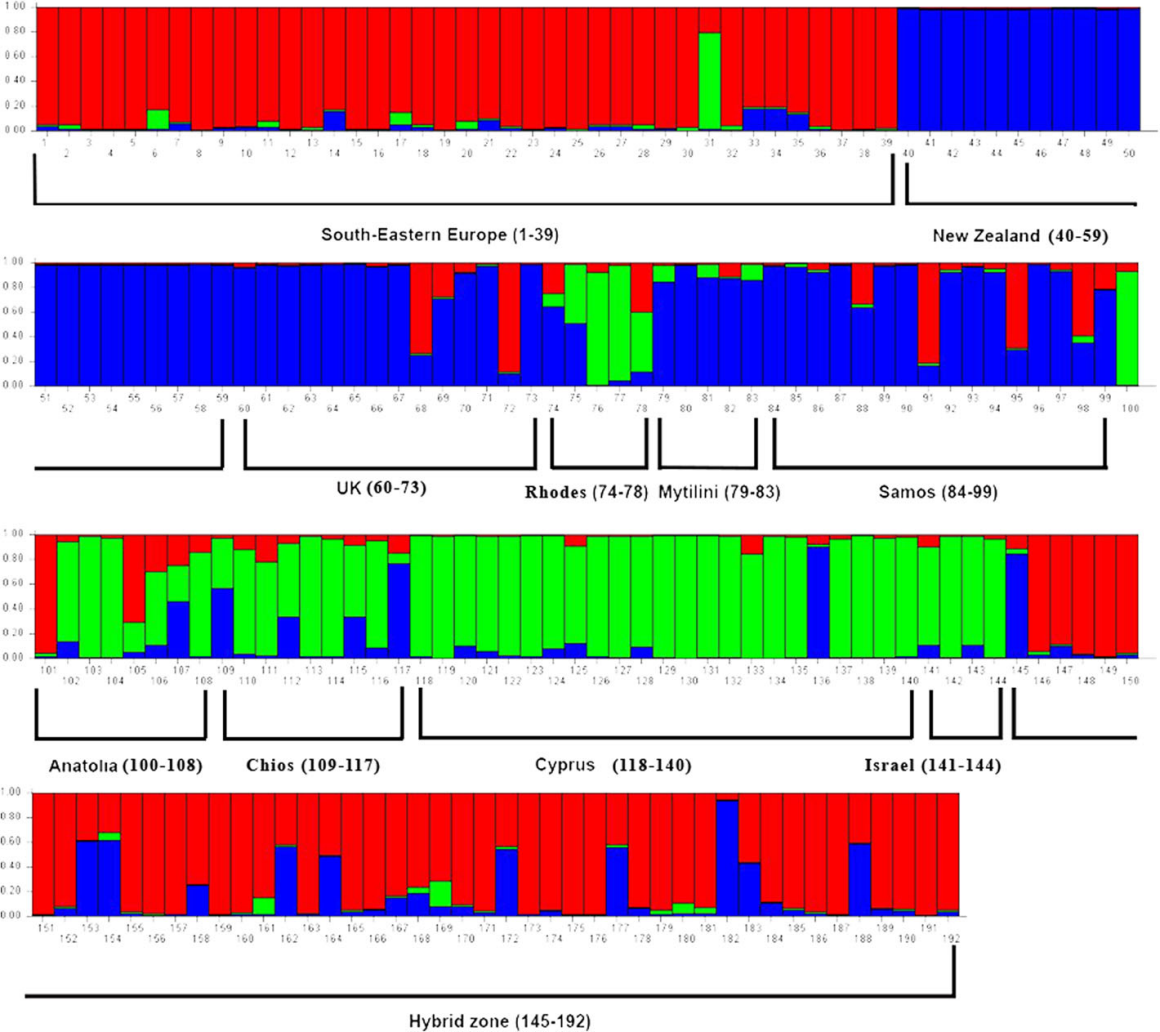
Samples’ assignments to three clusters, using the phylogeographic origin of the samples

Plotting the q-values of each individual resulted as seen in Fig. 5. The South-Eastern European samples (presented in red) and the Central European samples (in blue) show a discrete grouping from the Anatolian group (green colour). However, in this grouping, the samples from Mytilini and Samos appear to have the Central European pattern. Hybrid samples (145–192) are mostly assigned to the South-Eastern European group, with minor assignments to the Central European group while the Anatolian-type grouping is almost absent from the hybrid individuals (green color).

### Kinship analysis

Having considered that the hybrid population contains European (both Central and South-Eastern) and Anatolian haplotypes, we would expect the fullsib or halfsib relationships to be limited between individuals from the different clades in the case of pre-zygotic reproductive isolation between them. However, we detected halfsib relationships from all the different combinations (Central-South Eastern European, Central European-Anatolian, South-Eastern European – Anatolian). Since these halfsibs do not share the maternal lineage, as they carry a different mtDNA haplotype, we can conclude that these are paternal halfsibs (i.e. they have the same father).

## Discussion

To our knowledge, this is the first study of the species to combine the analysis of adaptive and non-adaptive variation in island and hybrid populations simultaneously. The importance of studying these populations is well documented elsewhere [1, 2, 7, 49, 50], since they represent perfect examples of isolation (islands) and mix of differentiated genomes (hybrid zones). The formation of these populations under dispersal/translocations was followed by the action of the forces that shaped their genetic pool, which led in the fixation of adaptive and non-adaptive variation as well. So, the adaptive variation that was generated and maintained (fixed) in the populations can be indicative for the populations’ history and local adaptation in the different habitats. On the other hand, non-adaptive variation that was generated probably by random mutations in the different populations can give useful insights in the phylogeography of the species, while the adaptive variations may fail to show a phylogenetic signal. One example is the MHC DQA locus, which reflects the absence of the phylogeographic signal: the pairwise *F*_st_ values do not follow the expected pattern of the phylogeographic status of the species (Table 2).

### Adaptive variation – DQA and mitochondrial loci

#### DQA exon 2

The different challenges each population had to face during the isolation in the distinct refugia was the selective force that shaped the DQA variation among the clades. This is reflected by the diversity in allele distribution in DQA gene between the Anatolian and European populations; they had to challenge different pathogens in their refugia, so specific alleles were favored by selection in each population. The overlap of common alleles between the clades was found to be absent [33] or limited (this study, Table 1). This overlap could also be a result of human-mediated translocations in the specific islands. However, even in common alleles, the frequencies showed a significant difference between the clades. These differences are highlighted in pocket variant frequencies as well as in combinations of pockets found in each allele (Table 3). Interestingly, the differences are not only observed between clades but also among populations belonging to the same clade (e.g. Pocket 1 variant YHQFWA is present in Samos and Cyprus, but totally absent from the rest, Pocket 6 variants NNTEN and NNTAG are only present in Chios and Cyprus). The same pattern of differentiation is observed in pocket combinations: unique combinations are observed in specific populations (YHQFWA/NNTEN/YNILR, YHQFWA/NNTAG/YGIMR in Cyprus population, YHQFWA/NNTAN/YNILR in Samos). Furthermore, these combinations are not only unique in specific populations, but are also absent from their presumably parental population of Anatolia [33]. So, the introduction of random mutations may have played a role in the appearance of the specific alleles and later on they were positively selected in order to be fixed in these territories. However, positive selection analysis did not reveal any PBR residues that are positively selected, since codon 8 does not contribute to the formation of any pocket. This codon however is in proximity with a codon forming the pocket 1 (codon 9) and with a codon forming pocket 6 (codon 11). There is a number of studies concerning positive selection on DQA genes, that failed to find evidence for positive selection on cotton rat (*Simgodon hispidus)* and the European woodmouse (*Apodemus sylvaticus)* [51, 52]. Also, a large proportion of codons under positive selection are positioned very close to PBR codons, as a consequence of inferring PBR residues of DQ genes from the three-dimensional structures of DR genes. The absence of positively selected residues in DQ genes may be a result either of a different assembly of DQ genes from the one inferred or by a weaker selection pressure in these genes [53]. This hypothesis is also supported by our finding of 6 codons under negative selection, which may be a result of different selecting pressures acting on DQ genes. Interestingly, there is a variety of positions identified in other species to be positively selected (codons 47, 55, 56, 68, 69, 76 and 79 in species from a variety of orders, e.g. Primates (*H.sapiens*), Rodentia (*M.musculus*), Lagomorpha (*O. cuniculus*), Cetartiodactyla (*B. taurus, O. aries*), Carnivora (*C.familiaris*)). These positions that are recognized in a variety of species as positively selected might have functional implication in antigen presenting process [53]. The remodeling of PBR positions from DQ specific crystal structure information could shed the light on this phenomenon.

Comparing the values of H_obs_ and H_exp_, we can notice the extremely low values of H_obs_ in all populations under study. These results are in contrast with the overdominance (the heterozygote advantage), which is believed to be one of the factors contributing to the high levels of MHC variation. This may be a result of a failure to amplify the second allele in the PCR reaction, so we get a pattern of homozygosity in SSCP pattern, or due to selecting only the exon 2 of the gene, where the samples appear to be homozygous, while they are heterozygous at other exonic regions. However, repeating the PCR/SSCP method or sub-cloning the PCR products and further sequencing them, did not change the results of the analysis.

Interestingly, H_obs_ values range from 0 (in Rodos population) to 0.33 in Mytilini population, with a weighted mean value of 0.18, while the continental populations from the study of Koutsogiannouli and her colleagues revealed a mean value of H_obs_ of 0.31 for the Greek haplogroup, 0.42 for the Central European and 0.42 for the Anatolian haplogroup. This reflects a signal of the founder effect: small island populations are created either by translocations (e.g. New Zealand, [20]) or by natural dispersal through temporal land bridges (e.g. Cyprus, [19, 20]) and a small proportion of the genetic diversity of the parental population is present in the newly formed island population, leading gradually to homozygosity through inbreeding and through the action of genetic drift, which is also responsible for the fixation of the newly found alleles in the island populations, which are totally absent in their parental populations.

Another example of adaptive variation is the genetic variance of CR under study among the different territories. The mitochondrial DNA, once considered neutral in terms of selection, is now believed to be under purifying selection [54]: protein-coding genes are encoding for subunits of the oxidative phosphorylation. Any mutations leading to impairment of the complexes of the OXPHOS would lead to reduction of the energy produced, having a major effect on individual fitness and reproductive success. Also, the CR is harboring essential items for mtDNA’s replication and expression. According to the above, the CR of the mtDNA may not be under selection per se but it is in high Linkage Disequilibrium with genes under selection, since the mtDNA is transmitted as a whole, due to the absence of recombination events. Traces of selection in the species’ mtDNA have been documented elsewhere [26].

In our populations, there is a clear phylogeographic signal in the CR (Fig. 2); the European haplotypes are grouped together and separately from the Anatolian haplotypes in the constructed phylogenetic tree. This could be explained by two, not mutually exclusive, hypotheses: CR is showing an elevated rate of fixing mutations since it is the only non-coding area of the mitochondrial DNA, making it more susceptible to mutations (this study, [26]) and is used for evolutionary studies in many animal species (e.g. Cattle, [55], European brown hare, [20]). There is also evidence for length variation along with sequence variation in the CR of certain species [56, 57]. On the other hand, the functional role of the CR described above, could lead to the assumption that this particular region is coevolving/coadapting with the cooperating nuclear genes, so the most adapted combination is favored in terms of effective replication and expression of mtDNA [58–60] under specific environmental conditions.

Concluding, the CR is affected by high mutation rate and possibly by coevolution patterns governing the relations between the two separate genomes, leading to locally adaptive variants, specific for each clade of the species.

It is noteworthy that CR variation is not only influenced by the population localization (mainland, island) but also by the clade of origin. In this study, we detected a higher rate of haplotypes per sample in mainland populations when compared to island populations (0.76 to 0.24 respectively, Table 7). However, after having considered the data of Stamatis and his colleagues (2009), the mainland populations of Anatolia exceeded a higher rate compared to mainland populations of Europe (0.48 to 0.21), while the Anatolian islands exceeded also a higher rate compared to the European islands (0.26 to 0.20) and to the mainland European populations (0.26 to 0.21). These observations are in accordance with the data obtained with mtDNA and allozyme markers [19, 61] and are probably caused by the biogeographic position of Anatolia, which is characterized by few environmental barriers for gene flow during the Pleistocene and Holocene epochs for many mammalian species [62, 63]. Also, the density of the sampling could be a factor for the variation, since the samples from the mainland Anatolian populations are limited compared to the samples from the mainland European populations (45 vs 751 in this study and [21]). A more extended sampling in the Anatolian populations might have lowered the intrapopulation differentiation levels.

**Table 7:** Total number of mutations and haplotypes per locus

Genomic Region	Number of mutations	Number of haplotypes	Geographic grouping
Control Region (CR)	79	53	Yes
tRNAs	8	10	No
Cytb	33	23	Partial
DQA exon 2	37	12	No

### Non-adaptive variation – Microsatellites

Concerning neutral variation, the study of the six microsatellite loci, resulted in the expected outcome, if we consider the phylogeographic status of the species. Using Evanno’s estimation of the real K of the populations, we calculated the number of discrete populations to be equal to 3, which corresponds to the distinct phylogenetic haplotypes of the species. The downstream analysis of the samples distribution and assignment revealed some discrepancies in particular island samples (e.g. Rodos, Mytilini), which may be a result of human-mediated translocations. However, when we studied variation in the level of mtDNA, these samples showed a clear signal of belonging to the Anatolian clade, as was revealed by the phylogenetic tree constructed by the CR as well as by the restriction pattern of the mitochondrial Cytb gene. We believe that these ambiguities are a result of human-mediated translocations of male individuals; human-mediated because there is no land bridge connecting the Central Europe to these islands and male individuals, since the mitochondrial clade remains of the Anatolian origin.

Tests for HWE revealed some specific population-locus combinations that deviated from the expected equilibrium. These deviations may be explained by the homozygote excess, as revealed by the Wright’s *F*_is_ values. Furthermore, null or rare alleles may be responsible for the deviations. According to Hale and her colleagues [64], there is a need for 25 to 30 samples per population to estimate the allele frequencies and the heterozygosity range of each population. In our study, this criterion is not met in specific populations with limited sampling, which affects the estimation of the frequencies which are used in the HWE models. Having taken these factors into account, we decided to kep all six markers for the downstream analysis.

The continental populations showed a higher level of H_obs_ compared to island populations (0.55 vs 0.34 respectively), which is a result of the creation of island populations under the founder effect and the action of genetic drift. Also, it is notable that in all six loci, island populations appear to have alleles that are absent from the parental terrestrial populations. Random mutations may be the “creative force” for these alleles, which remain to the population and establish more easily than the newly created DQA alleles, because of the selective neutrality of these nuclear loci. Pairwise comparison of the 3 groups that were formed in our analysis with Structure showed that the groups that comprised the South-Eastern European samples and the Central European samples had the minimum difference (0.11), while the groups South-Eastern European versus Anatolian and the Central European versus Anatolian showed an elevated differentiation (0.13 and 0.19 respectively). Samples from the hybrid zone, despite carrying all the possible mitochondrial haplotypes, show the South-Eastern European pattern as the dominant pattern in their grouping, with some particular individuals to show an intermediate pattern among the Central European and the South-Eastern European. Anatolian pattern is almost absent from the profiling of the samples of the hybrid zone, so we can hypothesize that the hybrids are not F1 hybrids, where we would expect an intermediate pattern in the samples. The SE European haplotype is in geographical proximity with the territory, so we can assume that it is the prevalent one in the specific area through random dispersal of individuals. Mitochondrial haplotypes of the other two groups may be present through the maternal lineage for a prolonged period of time, but their correspondent nuclear loci seem to decrease over time. The decline in their frequency may be a result of their limited dispersal along with the limited fitness of hybrids carrying an admixture of different mitochondrial and nuclear counterparts. Furthermore, when performed kinship analysis, we detected halfsib relationships among individuals carrying different mitochondrial haplotypes (e.g. Anatolian – SE European or Anatolian – Central European), meaning that these individuals share the paternal lineage. Thus, this is another indication that the clades do not only mix geographically in this territory but they also reproduce and produce offspring. Considering the fact that the lineages meet and mate but there is no introgression between them, we can hypothesize that there is a genetic barrier in the gene flow since there are no geographical barriers that could halt the translocations. This hypothesis can also be supported by our previous results [24, 26], where we detected that the OXPHOS genes, nuclear and mitochondrial, show an elevated rate of evolution between the clades of the species, that can cause incompatibilities in the adaptation and the reproduction of the species, when differentiated populations meet and mate in the case of hybrid zones. This phenomenon has also been observed in other species when differentiated populations were crossed to study the effect of the OXPHOS evolution rate in reproductive isolation (e.g. *Tigriopus californicus*, [65, 66], *Drosophila,* [67]).

Another possible explanation for this pattern of introgression is the outcome of the meta-analysis of Petit and Excoffier [68], which concluded that species with male-biased dispersal, like many mammals (and the species of *L.europaeus* among them [69, 70] appear to have an introgression biased towards mtDNA. Furthermore, according to Petit and Excoffier, the organelle genomes (and the respective markers like CytB, which assigns the samples to the haplogroups) introgress more easily than the nuclear genomes, because they are less likely to hitchhike with a region under selection, that could limit the introgression or even prevent it. A more comprehensive multilocus study of the adaptive variation in the hybrid zone population and in populations with ambiguities between their mitochondrial and nuclear background (e.g. Mytilini), using genomics approaches, would give useful insight on the population composition regarding their current condition and could infer some conclusions on population history and dynamics, which would be used on future approaches for the conservation and the management of the local populations, since the restocking operations is a common practice used worldwide for the species, especially for hunting purposes. The genomics approach could reveal the levels of genetic mixture of the clades in the nuclear level as well, so that we could detect incompatibilities between the cooperating genes of the two genomes by detecting unusual combinations of alleles in specific genes.

## Conclusions

Our multilocus phylogeographic analysis of European brown hare populations from islands allowed us to test the effect of the founder effect on these populations in terms of genetic polymorphism, compared to mainland populations. We found evidence that island populations show a lower level of genetic polymorphism but the geographic origin is not the only decisive factor, since the clade of origin seems to contribute as well in the levels of the genetic polymorphism of the island populations. Concerning the hybrid population, we confirmed the mtDNA introgression in the hybrid zone which was not followed by the nuclear DNA introgression of the invasive haplotype (the Anatolian) and we provided a possible explanation based on previous studies of the species and in a theory developed by Petit and Excoffier [60] that concerns species with male-biased dispersal. According to this theory, the introgression is biased towards mtDNA. Furthermore, using these results of introgression along with data from other studies [20, 21, 23] we can conclude that the Quaternary played a major role in the shaping of the phylogeographic patterns of the species, by isolating populations in distinct refugia, leading to genome differentiation and genomic conflicts observed nowadays.

## Methods

### Sample collection and assignment

**Fig. 6:**
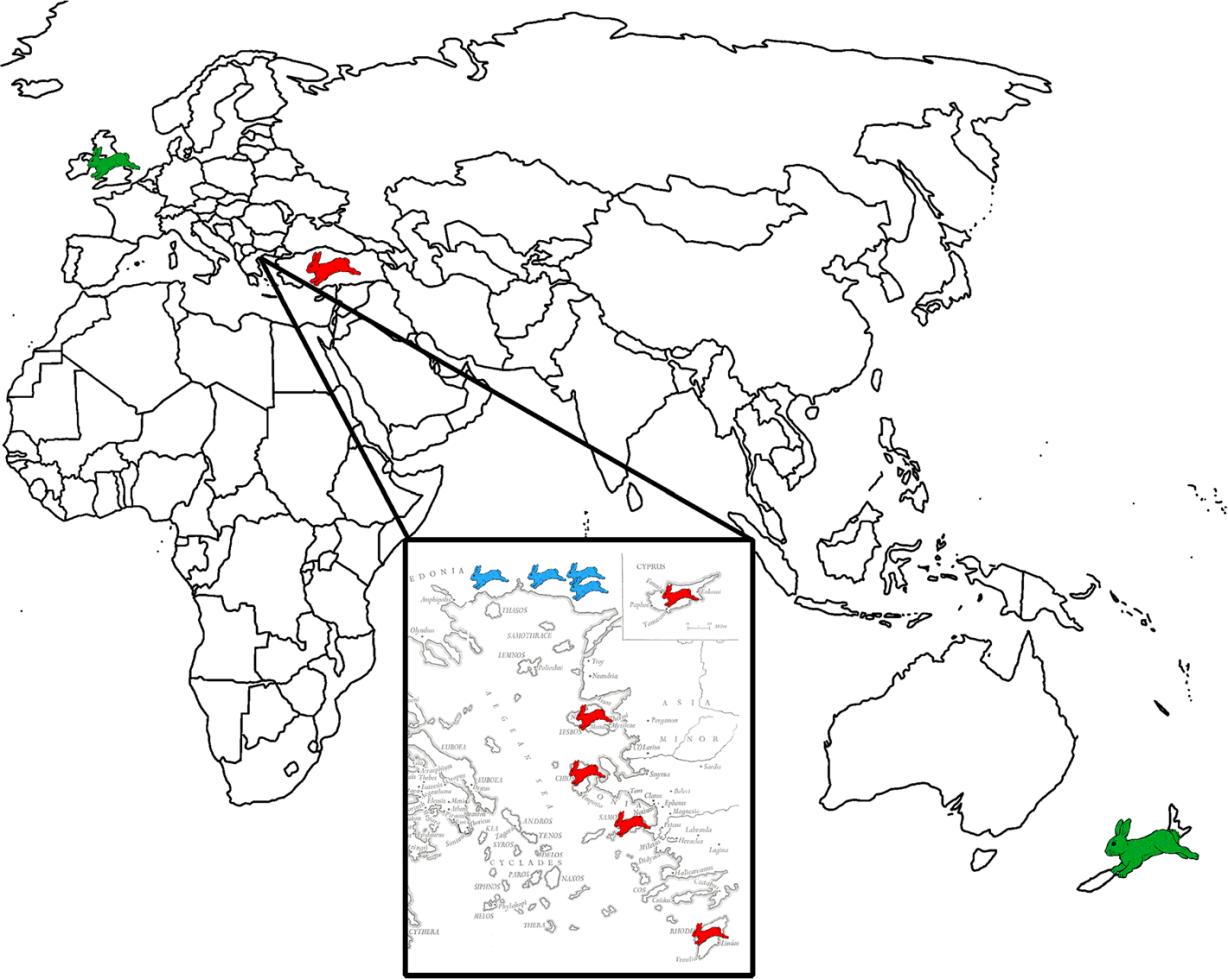
The sampling locations. The coloring is in accordance with the clade of the species present in each location (Green for the European Lineage, Red for the Anatolian)

One hundred ninety-two samples (192) were collected during 2000–2016 from hunting associations. Sampling areas are shown in Fig. 6. DNA extraction was performed using Invitrogen Kit (Invitrogen, Carlsbad, CA) according to the manufacturer’s protocol. The samples were assigned to the three different phylogeographic groups, using the method described in Stamatis et al. [39]: (a) the south-eastern European; (b) the central European; (c) the Anatolian.

### PCR amplification

The primers sets used for the analysis were: for DQA exon 2 DQAF:5′- TCATCAGCTGACCACGTTGG-3′ and DQAR:

5’-GACAGCAGCAGTAGAGTTGG-3′ (a 231-bp long amplified region) [71], LepCyb2L: 5’-GAAACTGGCTCCAATAACCC-3′ and LepD2H: 5’-ATTTAAGAGGAACGTGTGGG-3′ for the mitochondrial region (a 1200-bp long amplified region, comprising the partial CytB, the tRNA^Thr^ and tRNA^Pro^ and the partial CR) [48]. DQA samples were further screened for variation by single strand conformation analysis (SSCP) and sequencing, according to the method described in Koutsogiannouli et al. [33]. The hybrid, the Anatolian and the UK populations were analysed in our previous study [28] so they were excluded from this analysis. More specifically, 5ul of the PCR product were mixed with 10ul of loading buffer (95% formamide), denaturated at 99^ο^ C for 10 min and immediately kept in ice to avoid re-hybridization of the clones. The samples were loaded in a 10% acrylamide gel and electrophoresed overnight at 220 V. The results were visualized using silver staining and the samples were grouped according to their electrophoretic profile. Representative samples of each profile were selected and cloned in a pGEM-T easy vector and transformed into *E.coli* DH5a competent cells. Using this approach, we could obtain both sequences from a heterozygote profile and a unique sequence from a homozygous profile. After blue/white screening, a number of clones were selected and grown in order to perform isolation of the plasmid DNA. The subsequent PCR-SSCP analysis provided all the alleles in hemizygous condition, so we could perform the sequencing analysis. Sequences were aligned in Bioedit 7.0 and refined by eye. Along with these data, we used data from our previous analysis [33] that used the same genomic region.

For the microsatellite analysis, we used 6 primer sets (sol08, sol30, sol33, Lsa1, Lsa6 and Sat2) [72–74]. DNA samples were shipped to BGI China, where the amplifications and the sequencing reads were performed in an ABI 3730XL. Raw data were imported to GENEMARKER, exporting the peak figure (FSA files) and site information sheet (Excel format).

### Data analysis

#### MHC locus

Genotype and allele frequencies for each population as well as genotypic differentiation between island populations were measured using GenePop 4.6. Expected and observed heterozygosities and pairwise *Fst* values among populations were estimated using the package adegenet which is implemented in R [75, 76], using individual data (genind option in package). Synonymous and non-synonymous substitutions were detected with MEGA 7. Traces of selection acting in this region were estimated with Datamonkey gateway [77] under the FEL, REL and SLAC methods with levels of significance *p* < 0.05. FEL and SLAC methods use Maximum Likelihood approaches to infer nonsynonumous and synonymous substitutions rates per base of a given alignment while REL tests for selection signatures for each branch of the phylogeny instead of testing for specific sites [78]. To estimate pocket differences between the different alleles, a custom script was written to extract the combinations of partial pockets 1, 6 and 9 encoded by this particular region. The custom script extracts the specific pocket positions of a given amino-acid alignment according to Bondinas et al. [79] and records unique pocket variants (pocket 1 variant for each allele etc) and a combination of all three pockets for each allele of the alignment (pocket1-pocket6-pocket9 for allele 1 etc). The exact positions of the alignment that correspond to the pocket positions were retrieved from the study of Koutsogiannouli and her colleagues [33]. The frequencies of the combinations per populations were also calculated and compared with the respective frequencies from a former study on mainland populations of the species [33].

#### Mitochondrial DNA region

The 1200 bp-long region was further split in the three different loci: the partial CytB region, the tRNA^Thr^ and tRNA^Pro^ region and finally the partial CR, by aligning it to the reference genome (Accession number: AJ421471). The total numbers of mutations in each of the three sub-divisions of the amplicon were calculated using MEGA 7. We used the BlastClust algorithm to extract the unique sequences among the samples (100% length coverage plus 100% similarity) and distributed each sample to the unique haplotypes derived from the program. The total number of mutations and haplotypes for each region is presented in Table 7. Using the unique haplotypes, we constructed a Maximum Likelihood (ML) tree with 100 bootstrap replicates using MEGA 7 software, to test for the potent phylogeographic signal of the haplotypes (Fig. 2). Additionally, we performed a network analysis, using the Minimum Spanning Network approach (epsilon = 0) implemented in the program PopART [80] to visualize the spatial relationships between the unique haplotypes.

#### Microsatellites analysis

Genotype construction for each sample was extracted using GeneScan with default parameters, using LIZ 500 as a marker (ThermoFisher Scientific). The sizes of the peaks were estimated by comparing the samples’ peak distance from the peaks of the LIZ 500 marker, which are of known sizes (ranging from 35 to 500). In order to round all allele sizes in an equivalent way, the program tandem was used, which uses an exhaustive search using the power function (transformed allele size) = a + b × (observed allele size)^c^ while optimizing parameters a, b and c so that rounding errors are minimal when rounding transformed allele sizes to integers that fit the expected nucleotide repeat patterns [81]. The first step in data analysis was to check for Hardy-Weinberg equilibrium across all population and loci combinations and for linkage disequilibrium for each pair of loci across all populations. For the purpose, we used the program Genepop 4.6 (available online at: genepop.curtin.edu.au) [82]. We considered all tests of all loci/locus pairs to be part of a single experiment, which produced n*m independent tests of HW (n: number of populations, m: number of markers of study) and m(m-1)/2 tests of Linkage Disequilibrium. The *p*-values of each test were compared to the corrected α for multiple testing using the sequential Bonferroni correction, according to Holm and Rice [83, 84] to check for departures from HWE or for loci with signs of LD. At some particular combinations of populations and loci (e.g. Mytilini for the marker Sol08, Chios for the marker Sol30), the HW test could not be performed (one allele per locus) so we excluded these from the correction of α. Furthermore, we computed the Wright’s coefficient of inbreeding (Fis) for every population and locus $\left({F}_{is}=\frac{Hexp\hbox{-} Hobs}{Hexp}\right)$ [85]. (Positive values of Fis are indicative of deficiency in heterozygosity while negative values are indicative for excess. The specific departures of HWE are shown in Additional file 2: Table S1 with the corresponding Fis values. Pairwise *p*-values for the LD testing are shown in Additional file 3: Table S2.

For the downstream analysis, we used two different approaches. In the first one, we assumed the number of populations to be the number of distinct geographic groups (corresponding to the sampling areas) and we calculated the allele frequencies, observed and expected heterozygosity for each population and each locus using the package adegenet, and the pairwise *F*_st_ values for every locus, using the program MSA 4.05 [86]. In the second approach, we avoided the use of the geographic origin of the samples as a distribution criterion for populations assignment, for two main reasons: firstly, for samples in proximity, we cannot assume genetic similarity due to undefined genetic barriers in gene flow (a phenomenon which is observed in European hare populations in the contact zone) and secondly, geographic separation is not always resulting in genetic differentiation [87]. For these reasons, we inferred the real number of populations (from now on referred as K) using the Structure program [88]. The true number of K is usually inferred using the maximum value of LnP(D), which stands for the posterior probability of the data for a given K. However, this method fails to detect the real K, since the LnP(D) tends to increase slightly for larger Ks until it reaches a plateau. Hence, we used the alternative method, plotting the second order of change of L(K) (Δ(Κ)) as described in the study of Evanno and his colleagues [87], using among others simulated datasets of island populations and contact zones. We ran 20 simulations for each value of K ranging from two to nine and the mean value of LnP(D) along with the variance was used for further analysis. The calculations of the Δ(Κ) require multiple steps: calculation of the mean likelihood L(K) over 20 runs for each K, calculation of the mean difference between successive likelihood values of K, L’(K) = L(K)-L(K-1), which corresponds to the rate of change for the likelihood function. The third step includes a calculation of the absolute value of the differences between two successive values ok L’(K), which corresponds to the second order rate of change of L(K) (L’(K)). Finally, the estimation of ΔΚ is the average mean of L’(K) divided by the standard deviation of L(K) [87]. By examining the plot K~ΔΚ (Additional file 6: Figure S1) the real K was inferred for our data to be equal to three (K = 3). Samples were divided in three groups using the Q-values, which are automatically calculated by Structure runs. Then, we set K = 3 in a single structure run with all the details implemented to plot the distribution of the generated Q-values of each individual. The Q values represent the cluster membership coefficient, i.e. the probability of the individual to be a member of a specific genetic cluster.

Additionally, to assess the deeper structure of the hybrid population and infer the relationships between individuals, we used the program Colony [89], which implements likelihood methods to infer kinship relationships between individuals (halfsib and fullsib, sharing one or both parents respectively) using multilocus genotype data along with information about species ploidy, mating systems and genotyping error rates.

## Additional files

Additional file 1: **Table S3.** Pocket frequencies per population. (PDF 23 kb)

Additional file 2: **Table S1.** Deviations from HQE and the respective Fis values. (PDF 21 kb)

Additional file 3: **Table S2.** Pairwise test for LD among marker pairs. (PDF 18 kb)

Additional file 4: **Table S4.** Number of alleles, observed and expected heterozygosities in six microsatellite markers. (PDF 74 kb)

Additional file 5: **Table S5.** Observed and expected heterozygosities in six microsatellite markers. (PDF 19 kb)

Additional file 6: **Figure S1.** The K-Δ(Κ) plot using the Evanno’s approach. The peak of the plot is for K = 3. (JPEG 32 kb)
