# Supplementary material for: Islands and hybrid zones: combining the knowledge from “Natural Laboratories” to explain phylogeographic patterns of the European brown hare
Source: BMC Evol Biol. 2019 Jan 10;19:17. doi: 10.1186/s12862-019-1354-y (PMC6329171; doi:10.1186/s12862-019-1354-y)
Supplement: Supplementary file 1 — Table S3. Pocket frequencies per population. (PDF 23 kb) [file 12862_2019_1354_MOESM1_ESM.pdf]

Additional table 3: Pocket frequencies per population

Pocket 1	N.Zealand	Samos	Rodos	Mytilini	Chios	Cyprus
**YHEFWR**	92.11	51.43	66.67	41.70	20.59	17.50
**YHLFWT**	5.26	0.00	0.00	25.00	0.00	2.50
**YHQFWT**	0.00	4.29	33.33	33.30	79.41	0.00
**YHEFWA**	2.63	0.00	0.00	0.00	0.00	0.00
**YHQFWA**	0.00	44.29	0.00	0.00	0.00	80.00
**Pocket 6**						
**NETAN**	97.37	51.43	66.67	66.67	20.00	20.00
**NNTAN**	2.63	48.57	33.33	33.33	0.00	0.00
**NNTEN**	0.00	0.00	0.00	0.00	25.00	25.00
**NNTAG**	0.00	0.00	0.00	0.00	55.00	55.00
**Pocket 9**						
**YNILR**	97.37	100.00	83.33	66.67	94.12	45.00
**YNIMR**	2.63	0.00	16.67	33.33	5.88	0.00
**YGIMR**	0.00	0.00	0.00	0.00	0.00	55.00
