# Supplementary material for: Islands and hybrid zones: combining the knowledge from “Natural Laboratories” to explain phylogeographic patterns of the European brown hare
Source: BMC Evol Biol. 2019 Jan 10;19:17. doi: 10.1186/s12862-019-1354-y (PMC6329171; doi:10.1186/s12862-019-1354-y)
Supplement: Supplementary file 2 — Table S1. Deviations from HQE and the respective Fis values. (PDF 21 kb) [file 12862_2019_1354_MOESM2_ESM.pdf]

Additional table 1: Deviations from HQE and the respective Fis values

Population	Marker	p-value of HWE test	α	Fis
Samos	Sol30	0.0003	0.005	0.40
Cyprus	Sol30	0	0.005	0.45
Cyprus	Sol33	0	0.005	0.72
Cyprus	Lsa1	0.0013	0.004	0.39
Greece	Lsa6	0	0.007	0.61
Greece	Sat2	0	0.005	0
New Zealand	Sat2	0.0003	0.005	0.24
UK	Sat2	0	0.005	1
Cyprus	Sat2	0	0.005	0.32
