# Supplementary material for: Islands and hybrid zones: combining the knowledge from “Natural Laboratories” to explain phylogeographic patterns of the European brown hare
Source: BMC Evol Biol. 2019 Jan 10;19:17. doi: 10.1186/s12862-019-1354-y (PMC6329171; doi:10.1186/s12862-019-1354-y)
Supplement: Supplementary file 3 — Table S2. Pairwise test for LD among marker pairs. (PDF 18 kb) [file 12862_2019_1354_MOESM3_ESM.pdf]

Additional table 1: Pairwise test for LD among marker pairs

Pair of markers	P-value	α
Sol08 & Sol30	0.800	0.0035
Sol08 & Sol33	0.476	0.0035
Sol30 & Sol33	0.161	0.0035
Sol08 & Lsa1	0.695	0.0035
Sol30 & Lsa1	0.706	0.0035
Sol33 & Lsa1	0.660	0.0035
Sol08 & Lsa6	0.593	0.0035
Sol30 & Lsa6	0.511	0.0035
Sol33 & Lsa6	0.241	0.0035
Lsa1 & Lsa6	0.664	0.0035
Sol08 & Sat2	0.714	0.0035
Sol30 & Sat2	0.915	0.0035
Sol33 & Sat2	0.146	0.0035
Lsa1 & Sat2	0.033	0.0035
Lsa6 & Sat2	0.877	0.0035
