# Supplementary material for: Islands and hybrid zones: combining the knowledge from “Natural Laboratories” to explain phylogeographic patterns of the European brown hare
Source: BMC Evol Biol. 2019 Jan 10;19:17. doi: 10.1186/s12862-019-1354-y (PMC6329171; doi:10.1186/s12862-019-1354-y)
Supplement: Supplementary file 4 — Table S4. Number of alleles, observed and expected heterozygosities in six microsatellite markers. (PDF 74 kb) [file 12862_2019_1354_MOESM4_ESM.pdf]

Additional table 4: Number of alleles, observed and expected heterozygosities in six microsatellite markers

		Greece	N.Zealand	UK	Rhodes	Mytilini	Samos	Anatolia	Chios	Cyprus	Israel	Hybrid Zone
Solo8	H_{obs}	0,87	0,75	0,43	0,20	0,00	0,25	0,88	0,38	0,77	0,75	0,81
	H_{exp}	0,80	0,56	0,67	0,73	0,00	0,34	0,96	0,55	0,82	1,00	0,77
	Alleles	8	3	4	3	1	3	7	3	6	5	7
Sol30	H_{obs}	0,97	0,70	0,57	0,00	0,00	0,31	0,89	0,00	0,45	0,00	0,77
	H_{exp}	0,92	0,63	0,54	0,50	0,40	0,53	0,79	0,00	0,83	0,67	0,83
	Alleles	18	5	5	2	2	4	7	1	10	2	17
Sol33	H_{obs}	0,46	0,55	0,00	0,00	0,00	0,00	0,33	0,00	0,19	0,00	0,42
	H_{exp}	0,67	0,61	0,36	0,83	0,60	0,00	0,48	0,60	0,68	0,67	0,44
	Alleles	7	4	2	3	2	1	3	2	6	2	6
Lsa1	H_{obs}	0,82	0,40	0,50	0,25	0,60	0,50	0,50	0,50	0,48	0,75	0,69
	H_{exp}	0,76	0,54	0,65	0,71	0,88	0,67	0,47	0,79	0,79	0,88	0,70
	Alleles	7	3	4	3	4	5	4	6	8	3	6
Lsa6	H_{obs}	0,21	0,00	0,07	0,00	0,00	0,06	0,67	0,67	0,12	0,00	0,21
	H_{exp}	0,53	0,00	0,15	0,67	N/A	0,07	0,81	0,67	0,12	0,00	0,53
	Alleles	4	1	3	2	1	2	4	2	2	1	2
Sat2	H_{obs}	0,04	0,63	0,00	0,43	0,50	0,00	0,56	0,67	0,67	0,39	0,50
	H_{exp}	0,04	0,84	0,27	0,79	0,75	N/A	0,87	0,92	0,98	0,80	0,92
	Alleles	13	2	9	2	1	6	7	7	10	4	17
