# Supplementary material for: Islands and hybrid zones: combining the knowledge from “Natural Laboratories” to explain phylogeographic patterns of the European brown hare
Source: BMC Evol Biol. 2019 Jan 10;19:17. doi: 10.1186/s12862-019-1354-y (PMC6329171; doi:10.1186/s12862-019-1354-y)
Supplement: Supplementary file 5 — Table S5. Observed and expected heterozygosities in six microsatellite markers. (PDF 19 kb) [file 12862_2019_1354_MOESM5_ESM.pdf]

Additional table 5: Observed and expected heterozygosities in six microsatellite markers

Populations	Sol08		Sol30		Sol33		Lsa1		Lsa6		Sat2	
	Hobs	Hexp	Hobs	Hexp	Hobs	Hexp	Hobs	Hexp	Hobs	Hexp	Hobs	Hexp
**Anatolian**	0.66	0.80	0.39	0.90	0.14	0.69	0.45	0.76	0.25	0.46	0.54	0.87
**Central European**	0.49	0.70	0.46	0.66	0.19	0.42	0.50	0.70	0.04	0.04	0.30	0.73
**SE European**	0.83	0.78	0.88	0.89	0.45	0.60	0.75	0.72	0.14	0.33	0.72	0.87
