# Supplementary figures and images for: Islands and hybrid zones: combining the knowledge from “Natural Laboratories” to explain phylogeographic patterns of the European brown hare
Source: BMC Evol Biol. 2019 Jan 10;19:17. doi: 10.1186/s12862-019-1354-y (PMC6329171; doi:10.1186/s12862-019-1354-y)

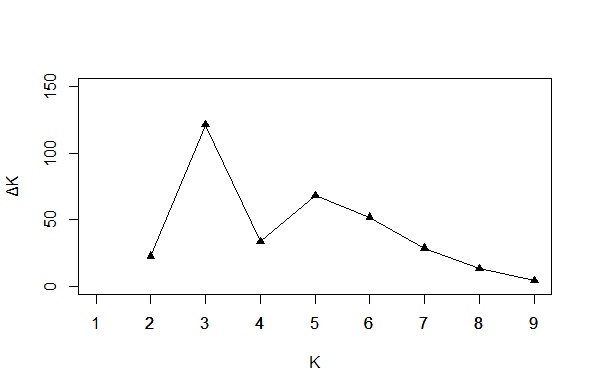

Supplement: Supplementary file 6 — Figure S1. The K-Δ(Κ) plot using the Evanno’s approach. The peak of the plot is for K = 3. (JPEG 32 kb) [file 12862_2019_1354_MOESM6_ESM.jpeg]
